# Presentation Accuracy of the Web Revisited: Animation Methods in the HTML5 Era

**DOI:** 10.1371/journal.pone.0109812

**Published:** 2014-10-10

**Authors:** Pablo Garaizar, Miguel A. Vadillo, Diego López-de-Ipiña

**Affiliations:** 1 Deusto Institute of Technology, DeustoTech., Universidad de Deusto, Bilbao, Spain; 2 Department of Experimental Psychology, University College London, London, United Kingdom; University of Cape Town, South Africa

## Abstract

Using the Web to run behavioural and social experiments quickly and efficiently has become increasingly popular in recent years, but there is some controversy about the suitability of using the Web for these objectives. Several studies have analysed the accuracy and precision of different web technologies in order to determine their limitations. This paper updates the extant evidence about presentation accuracy and precision of the Web and extends the study of the accuracy and precision in the presentation of multimedia stimuli to HTML5-based solutions, which were previously untested. The accuracy and precision in the presentation of visual content in classic web technologies is acceptable for use in online experiments, although some results suggest that these technologies should be used with caution in certain circumstances. Declarative animations based on CSS are the best alternative when animation intervals are above 50 milliseconds. The performance of procedural web technologies based on the HTML5 standard is similar to that of previous web technologies. These technologies are being progressively adopted by the scientific community and have promising futures, which makes their use advisable to utilizing more obsolete technologies.

## Introduction

Computers have been an indispensable tool for scientific research since the advent of computer science, not only because of their computing power, but also due to their ability to present multimedia information. Even prior to the popularization of graphical user interfaces, computers have been used to present visual stimuli in tachistoscopic applications subject to strict time constraints. The success of the Web as a primary medium of communication facilitated the creation of the first experimental applications based on web technologies [1].

Given the great diversity of web technologies, web user-agents, and existing operating systems, there is some controversy about the suitability of using the Web for these objectives. For this reason, several studies have analysed the accuracy and precision of different web technologies in order to determine their limitations. However, existing work is not comprehensive (e.g., GIF and Silverlight have not been explored thoroughly), and did not examine different options for controlling display in HTML5 (e.g., WebGL, CSS Animations). Therefore, an update seems necessary [2], [3]. The aim of this paper is twofold: (1) to provide an update of the extant evidence about presentation accuracy and precision of the Web in the presentation of multimedia stimuli, and (2) to extend these studies to HTML5-related technologies which have remained untested. The results of the present study have implications for researchers interested in developing online experiments where stimuli have to be presented under strict timing requirements (e.g., subliminal priming [4], [5]) or reaction times have to be measured with a high degree of accuracy and precision [6], [7].

### Declarative and procedural animations

Extant technologies for the accurate presentation of visual stimuli in web applications can be broadly divided in two different categories: those based on declarative animations and those based on procedural animations. The former focus on what should happen, while the latter describe how the desired goal will be achieved. Both approaches have advantages and disadvantages.

Declarative animations allow developers to define the requirements of the animation and forget about the low-level implementation details needed to meet them. However, there is no warranty of compliance. This is, if developers set up animations with requirements that cannot be met by the system (e.g., declaring a 10-ms colour transition to a device with a 50-ms timer granularity), final results may differ significantly from the original design. To create a declarative animation, developers define a set of keyframes and the animation engine (the browser in the case of web animations) generates all the steps between them. Listing 1 shows an example of a declarative animation where the initial and final keyframes are defined.

Listing 1. *Example of a declarative animation using SVG and SMIL. A 100 x 100 px red rectangle's width will be expanded from 100 to 500 px in 4 s, with a initial delay of 1s.*

  <?xml version = "1.0" encoding = "UTF-8"?>

  <svg xmlns = "http://www.w3.org/2000/svg">

   <rect id = "box" x = "200" y = "200" width = "100" height = "100" fill = "red">

    <animate attributeName = "width" begin = "1s" dur = "4s" from = "100" to = "500"/>

   </rect>

  </svg>

In procedural animations, objects are animated by a procedure (a script), not by defining keyframes. Developers can choose between different approaches to code the desired animation depending on the particular features of the available software and hardware. However, procedural animations are tightly coupled with the running environment (i.e., hardware, operating system, web user-agent versions, and so on) and might be affected by small changes to it.

### Web animations using classic web technologies

From its inception, the Web required complementary technologies to provide functionality not offered by HTML. HTML5 breaks this trend. The aim of this new standard is to provide HTML with enough features to create native web applications. We consider all pre-HTML5 web technologies as classic, even though they are still in use. In the following paragraphs, we give a brief overview of these web technologies.

#### GIF89a

Graphics Interchange Format (GIF) is a bitmap image format with animation features created by CompuServe in 1987. GIF89a is the version 89a (July, 1989) of this format. Advantages of GIF images include their small size and wide support of web user-agents. They have been extensively used since the beginning of the Web, and they are still common in web pages. GIF animations function independently of JavaScript.

Although this might be desirable for unattended animations, it means that GIF89a-based animations cannot be synchronized with other events. For example, the use of GIF animations is not recommended for applications that require high accuracy and precision. This is especially true when presenting several multimedia elements or registering the user interaction depending on the status of the animation (e.g., gather participants' response time after a target is presented in a priming experiment). The low granularity of the duration of each frame in a GIF89a animation is also problematic. GIF89a uses hundredths of a second instead of milliseconds to define durations [8]. This limitation is inexplicably masked by some GIF89a editing programs that allow specifying image durations in ms, even though they will be rounded to hundredths of seconds (e.g., GIMP – The GNU Image Manipulation Program). Similarly, some web user-agents automatically modify the intervals specified in GIF89a files if they are too demanding (e.g., in all versions of Microsoft Internet Explorer, intervals are rounded to 100 ms if they are lower than 50 ms).

#### Java

Java is both a programming language and a system for developing application software and deploying it in a cross-platform computing environment (the Java Virtual Machine, JVM). The Java programming language uses a syntax similar to C and C++. The main contribution of Java is the *“write once, run anywhere”* (WORA) approach: Java source code is compiled to Java bytecode, a hardware-independent binary code that can run on any platform with a JVM. The core benefit of Java is its portability and popularity in the field of software development. Java is undoubtedly considered one of the most popular programming languages.

With respect to the Web, Java has been used to create server applications (through servlets or Java Server Pages) and to deploy client applications (via Applets). Despite its success on all platforms, Java is gradually losing support from developers of web user-agents [9]. Recent security issues have resulted in fewer and fewer web sites relying on Java to extend the functionality of client-side web applications [10].

#### Flash

Flash is a technology for developing Rich Internet Applications (RIA). Using Flash, developers can handle different media types such as text, vector graphics and bitmap, audio, or video. Flash provides direct access to multimedia hardware like microphones or cameras, and it is being widely used in videoconferencing and multimedia streaming applications. People with no programming skills can program simple Flash applications using authoring tools such as Adobe Flash Professional. Professional developers can use the ActionScript language, a dialect of ECMAScript, to program complex Flash applications (e.g., experimental tasks for conducting behavioural studies). Once compiled, Flash applications run in a virtual machine known as Adobe Flash Player. This player can be installed for free as a plugin in the majority of web user-agents in many software platforms. The widespread deployment of Adobe Flash Player in users' browsers has ostensibly boosted Flash technology into the most popular RIA.

Its principal limitation is that it is not truly integrated into the web application, but embedded as an external object. As a result, Flash generates performance inefficiency and power consumption. Moreover, in 2011 Apple CEO Steve Jobs explained why they do not use Flash on iOS devices: *“Flash was created during the PC era – for PCs and mice. Flash is a successful business for Adobe, and we can understand why they want to push it beyond PCs. But the mobile era is about low power devices, touch interfaces and open web standards – all areas where Flash falls short.”* [11]. Shortly after, Adobe announced the end of Flash for mobile and TV platforms, focusing its efforts on the HTML5 standard [12]. Arguably, this popular technology has a bleak future.

#### Silverlight

Silverlight is Microsoft's answer to the success of Flash. Therefore, Silverlight is also a technology for developing RIAs that relies on a virtual machine to run them. The Silverlight virtual machine is available for the majority of web user-agents on Microsoft Windows and Apple Mac OS X. The Moonlight project provides unofficial Silverlight support for GNU/Linux, FreeBSD and other free operating systems. The main advantage of Silverlight over the aforementioned technologies is its integration with other development technologies based on Microsoft.Net. However, the lack of multiplatform support (officially, only for Microsoft Windows and Apple Mac OS X) and the popularity of a competing technology such as Adobe Flash, has relegated its use to scenarios where Microsoft technologies are used exclusively.

#### Native code

When the main goal is to maximize the accuracy and precision of animations, it might be tempting to rely on native code for those user-agents that allow that possibility. However, both Microsoft Active X [13] and Gecko NPAPI [14] show that granting execution privileges to native code from user-agents might not be a good security policy. Currently, their use is restricted to applications within corporation intranets where those security problems can be properly monitored and isolated. Furthermore, both technologies are limited to specific user-agents (Microsoft Internet Explorer in the case of Active X, and Gecko-based browsers, among which Mozilla Firefox is the most prominent example, in the case of NPAPI). Therefore, they are far from being multiplatform solutions. The third option for running native code, Google Native Client [15], aims to solve both problems, providing an execution environment automatically protected and supported by multiple platforms, but its development is still in the early stages.

### Web animations in the HTML5 era

HTML5 provides a wide variety of mechanisms to create animations. Web developers can use application programming interfaces (APIs) to create declarative animations such as Cascading Style Sheets (CSS) or Scalable Vector Graphics Animations (SVG) with Synchronized Multimedia Integration Language (SMIL), or procedural animations via SVG with JavaScript, HTML Canvas or WebGL. The well-known inaccuracy of standard JavaScript timers (setTimeout and setInterval) [16] can be avoided via the Using Timing Control for script-based animations API (requestAnimationFrame). Using the Timing control for script-based animations API, developers request animation updates to the browser, instead of trying to figure out when is the best moment to do it themselves. Considering that the browser keeps control of all the running animations using this API, it is in a better position to determine the optimal frame rate to run all the animations as smoothly as possible. In the following paragraphs, we give an overview of these web technologies.

#### CSS Animations

This API allows the animation of HTML document elements using CSS. Web designers and developers create a CSS animation declaring a set of keyframes with different transitions between them. The CSS Transitions API provides a way to define a transition timing function (e.g., linear, ease-in-out, cubic-bezier) to perform a transform of a CSS-styled HTML element. These two-dimensional (e.g., scaleX, skewY) or three-dimensional (e.g. rotate3d) transforms are defined using the CSS Transforms API. The main advantage of using CSS Animations (and related APIs to define CSS transitions and transforms) is to provide semantic information about the animation to the web user-agent (so they can then decide the most effective way to run the animation.) In addition, CSS Animations do not need JavaScript events to run. Leaving free the JavaScript event queue is a good practice to increase responsiveness of the web application. The main disadvantage of CSS Animation is its lack of flexibility for defining animations. CSS Animations are limited to a combination of the features offered by CSS Transitions and CSS Transforms. Furthermore, not all animation can be created declaratively. Finally, support for these new standards is still incomplete in mobile browsers.

#### SVG with SMIL

SVG is a XML dialect for creating vector images. Among its many features, it includes the ability to embed JavaScript or to use SMIL to create declarative animations. The advantages of using SMIL in SVG are similar to the advantages of using CSS animations: it provides semantic information about the animation, avoids JavaScript timers, and creates animations that can be used outside the browser. SVG with SMIL shares all the disadvantages of CSS Animations. In addition, support for SMIL in most modern browsers is incomplete. Despite the renewed interest in SVG due to HTML5, browser vendors have devoted more effort to comply with the CSS Animations standard than improving their support of SVG with SMIL.

#### SVG with JavaScript

JavaScript code can be seamlessly embedded into SVG files. Within a SVG file, JavaScript is able to change its properties, create new shapes or animate them over time. HTML5 allows the inclusion of SVG mark-up in HTML documents and interaction with other elements of the Document Object Model (DOM, a hierarchical representation of the structure and style of HTML documents). The main advantage of using SVG with JavaScript is the low consumption of computational resources when working at high resolutions, due to its vector nature. Even in procedural animations, SVG provides semantic information about the shapes that are animated. Therefore, browsers can optimize the display update process. Another benefit is the flexibility of combining SVG and JavaScript. As mentioned before, procedural animations can go beyond the possibilities offered by declarative animations. The main disadvantage of combining SVG and JavaScript is the poor performance of JavaScript timers. In addition, performance is severely affected as the number of shapes in SVG increases. Using it for animations that constantly change the objects in scene is not recommended.

#### Canvas

The HTML Canvas 2D Context API provides a blank canvas (a blank bitmap) to draw shapes, text, or images on it. Canvas contents can be easily modified applying transforms (scale, rotate, translate), compositing changes, or modifying shadow attributes. This HTML element has been essential to creating video games in HTML5. It offers good performance in complex scenes and features like dumping the content of the canvas to an img element. Among its drawbacks, two stand out: the dependence on JavaScript timers to update animations, and a progressive loss of performance as the size of the scene to render increases.

#### WebGL

WebGL enables a 2D/3D context for the HTML5 canvas element. WebGL specification is derived from OpenGL ES 2.0. WebGL provides a 3D immediate mode rendering API where OpenGL-like resources (textures, buffers, framebuffers, renderbuffers, shaders, and programs) are represented as DOM objects. The main advantage of WebGL is its good performance with 3D scenes. WebGL and OpenGL are very similar, allowing game developers to easily port their skills to the Web. Recent Graphics Processing Units (GPU) provide the 3D acceleration needed for an optimal experience with WebGL. There are two disadvantages of using WebGL: the complexity associated with 3D graphical programming, and the possibility that there is no 3D hardware acceleration. In this case, WebGL reverts to 3D software rendering, and the overall performance is greatly affected.

### Previous studies

In 2001, Schmidt assessed the accuracy of different web animation methods, including GIF89a-based animations [2], and estimated that intervals below 200 ms should not be defined in GIF89a. Technologies based on virtual machines, such as Java or Flash, have been widely used to control the presentation of audio-visual contents for longer than a decade now. Schmidt also assessed their accuracy and precision, concluding that time intervals were reliable only above 100 ms in Java and 250 ms in Flash. Given the subsequent improvements to both virtual machines, it is necessary to update that evaluation, as well as include less common technologies, such as Silverlight.

There is no information available about the accuracy of the execution of time intervals in SMIL under the most widely-used players regarding SVG with SMIL-based animations. These players include Apple's QuickTime Player, Microsoft's Windows Media Player, and RealNetwork's RealPlayer, as well as the web user-agents themselves, which can show SVG and SMIL animations either natively or via plug-ins.

The World Wide Web Consortium (W3C) is prioritizing CSS Animations. Although this specification has not reached the status of ”standard,” the most recent versions of web user-agents are already compatible with CSS Animations [17]. Perhaps because of this, the accuracy and precision of this technology has not been tested thoroughly. Most web developers have relied on JavaScript to generate procedural animations, mainly because it does not require installing any plug-in in the most popular web user-agents (i.e., Microsoft Internet Explorer, Mozilla Firefox, Google Chrome, Apple Safari, Opera).

Nevertheless, this solution is constrained by its limited execution speed. The performance of JavaScript interpreters has improved very little until the recent popularity of applications with sophisticated web interfaces (e.g., Google Maps, Gmail). Schmidt also included an evaluation of JavaScript procedural animations, and concluded that JavaScript should not be used for procedural animations with an interval lower than 120 ms. Adams compared procedural animations based on JavaScript with those based on Flash and he found differences between them, in favour of the latter [18]. However, during the last years we have witnessed a revolution in the development of new APIs within the HTML5 standard and their adoption by the main user-agent developers, particularly by Google Chrome and Firefox. Because of this, some authors have published updated results about JavaScript-based animations in terms of accuracy and precision [19], [20].

Regarding the accuracy and precision measuring the user interaction, several researchers have analysed a variety of web technologies, particularly Java and Flash. Eichstaedt developed a filter to discard unreliable measurements of reaction times in Java applets using a control thread with a timer to detect the influence of the operating system load in the measurement [3].

Keller, Gunasekharan, Mayo, and Corley proposed a filtering algorithm for inaccurate measurements – similar to the one of Eichstaedt – as part of their online experiment suite, WebExp, implemented in Java [21]. After filtering inaccurate measurements, they obtained standard deviations around 1 ms. Similarly, McGraw, Tew, and Williams compared the precision of reaction times obtained with Macromedia Authorware (a decreasingly popular authoring tool for the development of applications that could be run both natively or from a web user-agent) against E-Prime, a well-known, tachistoscopic software for experiment design [22]. After manipulating user-agents, system load and network traffic, they concluded that both technologies had similar precisions for animations with 150, 200, 250, and 1000 ms intervals, except under conditions of high system load. The results of Schmidt also pointed towards 100 ms as the lower threshold for reliable measurements in Authorware.

A study by Reimers and Stewart analysed the validity of Flash to measure reaction times accurately in online experiments [23]. They found that measurements based on Flash under laboratory conditions were 10–40 ms slower than those made by a native Linux application [24]. The difference was 30–40 ms greater when tests were conducted outside the laboratory. The following year, they assessed the mobile version of Flash (Adobe Flash Lite) and found the estimations of reaction times were substantially greater in the mobile version (60–80 ms) compared to its desktop analogue. Similarly, they observed that the distribution of these measurements varied in different devices [25]. Schubert et al. also found a 10–35 ms lag in response times gathered using their Flash-based experiment software (ScriptingRT) compared to those gathered using offline experiment software, such as E-Prime and DMDX [26]. Finally, Reimers and Stewart conducted a study on the presentation and response time measurement accuracy in Flash and HTML5/JavaScript web experiments. They found that Flash and JavaScript's presentation and response time measurement were similar within the same system, but they varied greatly across different hardware systems [20].

### Research rationale

The Web was born in a research centre, and it has been closely linked to the research world ever since. Researchers from various disciplines use the Web to conduct their experiments, and social scientists are especially interested in conducting experiments on the Internet. Compared with laboratory experiments, it is relatively easy to access large samples on the Internet. However, researchers have little control over the experimental conditions in which participants take part in online experiments. Online researchers do not know if the participants have been paying attention to the task or have correctly understood the instructions. The pioneers of Internet-based research often had to face a high level of scepticism in the evaluation of their studies. Therefore, they conducted numerous studies comparing social and psychological effects in the laboratory and on the Internet. Except for minor exceptions, the overall conclusion is that the advantages of performing online experiments outweigh their possible disadvantages [1].

Among the studies that arouse methodological concerns are the those that require precise and accurate presentation of stimuli and response time measurements. As previously mentioned, there are several studies on the suitability of web technologies to conduct online experiments within strict time constraints [27], [22]. However, there is a need to update the results and conclusions obtained in these studies due to the continuous evolution of web technologies. During the last decade, studies of the suitability of some web technologies such as Java or Flash have been properly updated. Other web technologies have not received the same attention and the available experimentation data is more than 10 years old. Some of the earlier drawn conclusions may not stand any longer. Therefore, an update of these measurements is necessary and thus performed in this work.

At the same time, the Web has evolved into the most prominent platform for developing applications. Nearly every service is available through the Web. The apparent simplicity of an architecture based on a small set of well-known methods has been able to cover a myriad of different scenarios. The maturity of the Web has been made possible by the latest technological advances in its infrastructure (i.e., on-demand scalable computing resources) and user-agents (i.e., fully-featured web applications players). However, HTML5 is not an isolated specification; it is related to a broad set of technologies that should be analysed in detail.

Although some researchers have previously studied the accuracy and precision of HTML5 applications, there is a dearth of comprehensive evaluations about the suitability of these web technologies at software level. Because they do not rely on the same timing mechanisms, it is not only necessary to conduct between-systems studies due to the heterogeneity of devices used by participants in online experiments, but also to analyse all the possibilities offered by HTML5 to create animations on the same system. There are combinations of HTML5 APIs recommended for some cases and discouraged in other cases. The main aim of this study is to assess the accuracy and precision of web technologies to create animations for experimental paradigms, taking into account the type of animation used (declarative vs. procedural) and the timers involved.

To achieve our goal, we have conducted three studies. In Study 1, we analysed the accuracy and precision of animations that use different timing mechanisms in Flash. In this study, each timer was evaluated in several consecutive tests to determine whether any degradation of accuracy and precision over time occurs. In Study 2, we analysed several classic (i.e., pre HTML5) web technologies with a dual purpose: 1) updating or providing new data on the accuracy and precision of web technologies not analysed in the last decade (GIF89a and Silverlight), and 2) repeating some of the previous studies on already analysed technologies (Java and Flash) to provide more evidence and a baseline to compare the results of our study with previously published results. Finally, in Study 3 we compared the combinations of modern web technologies (i.e., related to HTML5) to create declarative (CSS Animations, and SVG with SMIL) and procedural (SVG with JavaScript, Canvas, and WebGL) animations.

## Study 1: Analysis of timing mechanisms in Flash

The aim of Study 1 is to find out which of the combinations between timing mechanisms and frame rates available in Adobe Flash is the most accurate and precise. Once identified, we used this optimal timing mechanism and frame rate combination to create the Flash animations analysed in Study 2. Each combination was evaluated in several consecutive tests to see if any degradation of accuracy and precision as a function of time occurred.

### Methodology and apparatus

The potential delays that can take place when presenting visual content in a personal computer makes using the same computer to present content and assess its time accuracy unreliable. Ideally, an external system, fully equipped with the necessary sensors, should be used to register the precise moment in which the content is shown (*onset time*) and the moment in which is removed from the screen (*offset time*). For this reason, all our tests have been conducted using the Black Box Toolkit (BBTK) as an external measurement device [28], [29].

We decided to use an Apple computer to test each technology. This allowed us to compare the most popular operating systems (Microsoft Windows, GNU/Linux, and Apple Mac OS) in the same physical device. The computer was an Apple MacBook Pro A1211 with an Intel Core 2 Duo T7600 processor, an ATI Radeon Mobility X1600 graphics card, a SigmalTel 9220 A1 High Definition Audio sound card, and a 1400×900 pixel LCD monitor at 60 Hz. We tested the accuracy and precision of the visual display of this equipment in two separate tests. One of them used the six-hour version of E-Prime's RefreshClockTest, with favourable results. The other one was PsychoPy's timeByFrames, which also yielded favourable results.

We installed Microsoft Windows 7 Professional 32-bit edition with Service Pack 1 in the computer described above. We used the latest version of Google Chrome web user-agent that was available when the study was conducted (Google Chrome 17).

### Procedure

The following tests were based on a procedure similar to that of Schmidt [2]. We defined non-gradual black to white keyframe transitions. All the tests consisted of a 200×200 pixel animation placed at the centre of the screen. We tested two factors: the timing mechanism and the FPS rate of the Flash animation. For each case, five independent 60-second series were recorded, and only the first 100 samples of each series were analysed. Therefore, 500 samples were recorded for each combination.

We evaluated the following timing mechanisms: (1) loop, leaving the control of the animation in the hands of the loop parameter of the Flash player; (2) no-loop, resetting the animation after the last keyframe (gotoAndPlay(1)); (3) setInterval, by means of an aperiodic ActionScript timer (setInterval(changeBackground, 50)); (4) polling, checking actively whether it is time to change the background (see Listing 2); and (5) timer, controlling the animation through a Timer object (see Listing 3). We evaluated the following FPS rates: (1) 20 FPS, very frequent in banners and simple Flash applications (an update every 50 ms); (2) 60 FPS, the refresh rate of the screen (an update every 16.667 ms), and (3) 100 FPS, above the screen's refresh rate (an update every 10 ms). As mentioned before, we conducted five consecutive tests for each combination in order to discover any degradation of accuracy and precision as a function of time (i.e., during a ten-minute long test, the first 100 samples were collected at the beginning of the first minute, the second 100 samples at the beginning of the third minute, the third 100 samples at the beginning of the fifth minute, the fourth 100 samples at the beginning of the seventh minute, and the last 100 samples at the beginning of the ninth minute of the test). All the tests were conducted on Google Chrome 17 and Microsoft Windows 7 SP1, which are the most popular user-agent and operating system, respectively. Additionally, we only evaluated the 50-ms interval, as it marks a turning point in the quality of performance in all our preliminary tests. All tests are available for download at https://osf.io/6j3iz/.

Listing 2. *ActionScript code snippet to control an animation through polling.*

  addEventListener(Event.ENTER_FRAME, update);

  function update(event:Event) {

     var currentTime:int  =  getTimer();

     var newCurrentTime:int  =  getTimer();

     var dtInt:int  =  newCurrentTime - currentTime;

     while (dtInt <50) {

       dtInt  =  newCurrentTime - currentTime;

       newCurrentTime  =  getTimer();

     }

     changeBackground();

  }

Listing 3. *ActionScript code snippet to control an animation through a Timer object.*

  var myTimer:Timer  =  new Timer(5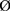);

  myTimer.addEventListener(TimerEvent.TIMER, changeBackground);

  myTimer.start();

### Results and discussion

Before analysing the results of our studies, we decided to compute missed frames, rather than measured timing errors (MTEs), for several reasons. Because the focus of these studies is the analysis of the accuracy and precision of several software mechanisms to create animations for the Web, timing errors attributable to hardware issues (i.e., rise and decay times of LCD displays) should not be considered as software errors. The same happens with limitations in measurement equipment (i.e., the BBTK photosensors do not provide a continuous analogue value, but rather a discrete digital one based on an adjustable threshold). Therefore, we decided to convert the MTE to missed frames using the formula: 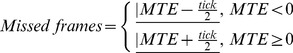
(1)


where 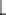 denotes the floor function.

A a missed frame represents a single screen refresh (i.e., 16.667 ms at 60 Hz) and it is constant across all conditions. Missed frames could be negative in animations where presentation times are at least one tick shorter than estimated duration (e.g., a 80-ms blank keyframe in a 100-ms interval animation).

**Figure 1:**
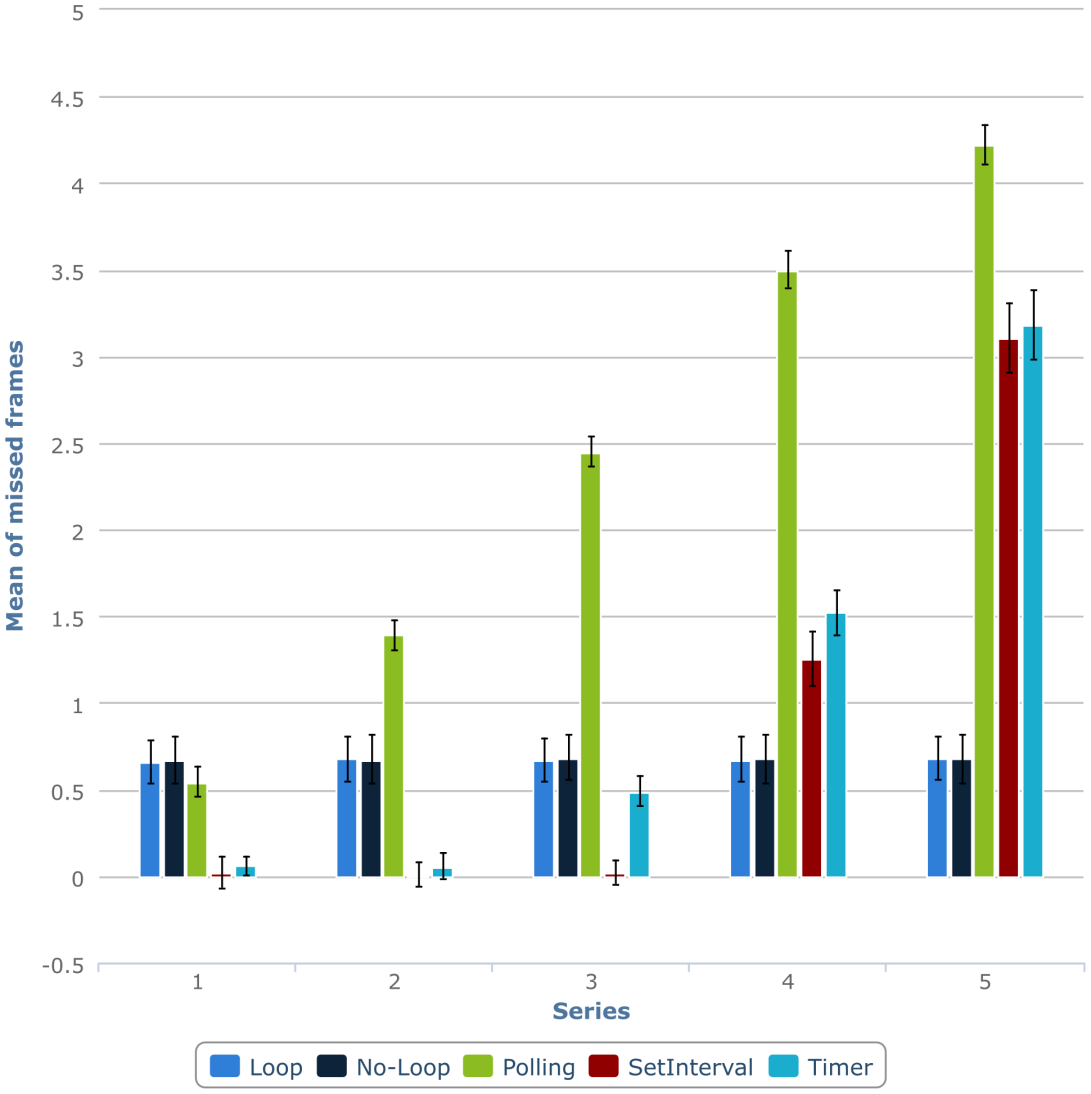
Number of missed frames per series for Adobe Flash animation using different timing mechanisms.

As shown in Figure 1, although the loop and no-loop timing options remain stable throughout the five series, the performance of the rest of timing mechanisms (setInterval, polling, timer) suffers a progressive degradation through the testing. To determine which factors have an impact in the number of missed frames, we analysed them with an analysis of variance (ANOVA) with Timer (loop, no-loop, setInterval, polling, and timer), FPS (20, 60, and 100 FPS), Series (1–5), and Colour (White vs. Black) as factors. Given the large number of measurements analysed (N = 7500), all main effects and interactions were significant. Consistent with our preliminary analysis, the main effect of FPS was relatively large, F(2, 7499) = 4201.554, p<0.001, η_p_^2^ = 0.533, confirming that there is a decline in performance when the number of FPS is lower (20) or higher (100) than the refresh rate of the screen. Interestingly, the main effect of Timer, F(4, 7499) = 2751.154, p<0.001, η_p_^2^ = 0.600, and the main effect of Series, F (4, 7499) = 3390.185, p<0.001, η_p_^2^ = 0.649, were also significant, showing that choosing a proper timing mechanism has a noticeable impact on performance.

The results of the ANOVA also revealed important interactions between some of these factors: The Timer x FPS x Series interaction, F(32, 7499) = 118.232, p<0.001, η_p_^2^ = 0.340, the Timer x FPS interaction, F(8, 7499) = 662.020, p<0.001, η_p_^2^ = 0.419, and the Timer x Series interaction, F(16, 7499) = 653.211, p<0.001, η_p_^2^ = 0.587, were all significant. Although significant, all other main effects and interactions had small effect sizes (all η_p_^2^<0.2).

Even ignoring the time degradation across series, the timing mechanisms that we have evaluated can be sorted from best to worse as follows: (1) loop (M: 0.67, SD: 1.087), (2) no-loop (M: 0.68, SD: 1.175), (3) setInterval (M: 0.88, SD: 1.658), (4) timer (M: 1.06, SD: 1.591), and (5) polling (M. 2.42, SD: 1.58). Therefore, we used the 60-FPS loop timing mechanism in all subsequent tests on Adobe Flash (see Study 2).

## Study 2: pre-HTML5 web technologies

The aim of Study 2 is twofold: 1) to provide new evidence on the accuracy and precision of animations created with web technologies not studied in more than ten years (GIF89 and Silverlight); 2) to recreate some of the previous studies on web technologies already analysed during the last decade (Java and Flash).

### Methodology and apparatus

The hardware for this study was the same as setup used in Study 1. Regarding the software, we used three web user-agents compatible with Microsoft Windows 7 Professional 32-bit edition with Service Pack 1: Internet Explorer, Google Chrome, and Mozilla Firefox. In each case, we used the latest version that was available when the study was conducted (Internet Explorer 9, Google Chrome 17, and Mozilla Firefox 10).

### Procedure

The procedure of Study 2 is also similar to that of Schmidt [2]. We defined non-gradual black-to-white keyframe transitions varying the duration of each keyframe with values of 500, 100, 50, and 16.667 ms (i.e., 30, 6, 3, and 1 tick at 60 Hz, respectively). All the tests consisted of a 200×200 pixel animation placed at the centre of the screen. For each case, five independent 60-second series were recorded, and only the 100 first samples of each series were analysed. Therefore, 500 samples were recorded for each combination of interval (500, 100, 50, and 16.667 ms), web technology (GIF89a, Flash, Java, and Silverlight), and user-agent (Internet Explorer, Google Chrome, and Mozilla Firefox).

GIF89a was tested using 200×200 pixel animations with a resolution of 72 points per inch (ppi). The animation consisted of two keyframes, one black and one white, shown for a specific interval. Given that this technology does not allow defining intervals shorter than 10 ms, these intervals were set to 500, 100, 50, and 10 ms in separate tests.

Adobe Flash was tested with loop-animations at 60 frames per second (FPS). Given that the refresh time is constant, we varied the number of black and white keyframes to obtain the desired intervals. Thus, the 16.667 ms animation consisted of just one black keyframe followed by one white keyframe, while the 500 ms animation consisted of 30 black keyframes followed by 30 white keyframes. The justification for this choice is explained thoroughly in Study 1.

Regarding Java, we used an applet with a single parameter defining the animation interval measured in ms. Therefore, we prepared four HTML documents with a 200×200 pixel applet and the appropriate animation interval (500, 100, 50, or 16.667 ms). This applet uses the scheduleAtFixedRate method from the ScheduledExecutorService interface, receiving as arguments a specific thread (Runnable interface), and initial delay, an interval, and a measurement unit (see Listing 4). The following setup was used: 1) the thread called the repaint method, which changes the applet's background; 2) the initial delay was set to 0; 3) the interval is taken from the applet's parameter; and 4) the time unit was fixed to ms (TimeUnit.MILLISECONDS). This produced a periodic change in the background colour.

Listing 4. *Java applet to run a simple procedural animation.*

import javax.swing.JApplet;

import java.awt.Color;

import java.awt.Graphics;

import java.awt.Dimension;

import java.awt.Image;

import java.util.concurrent.Executors;

import java.util.concurrent.ScheduledExecutorService;

import java.util.concurrent.TimeUnit;

public class Square extends JApplet {

  int speed, h, w;

  boolean isWhite;

  Graphics bufferGraphicsWhite;

  Image offscreenWhite;

  Graphics bufferGraphicsBlack;

  Image offscreenBlack;

  public void init() {

   speed  =  Integer.parseInt(getParameter("speed"));

   Dimension d  =  getSize();

   h  =  d.height;

   w  =  d.width;

   isWhite  =  true;

   offscreenWhite  =  createImage(w, h);

   bufferGraphicsWhite  =  offscreenWhite.getGraphics();

   bufferGraphicsWhite.setColor(Color.WHITE);

   bufferGraphicsWhite.fillRect(0, 0, w, h);

   offscreenBlack  =  createImage(w, h);

   bufferGraphicsBlack  =  offscreenBlack.getGraphics();

   bufferGraphicsBlack.setColor(Color.BLACK);

   bufferGraphicsBlack.fillRect(0, 0, w, h);

   try {

    ScheduledExecutorService executorService  =  Executors.newSingleThreadScheduledExecutor();

    executorService.scheduleAtFixedRate(new Runnable() {

@Override

     public void run() {

      repaint();

     }

    }, 0, speed, TimeUnit.MILLISECONDS);

   } catch (Exception e) {

    System.err.println("Applet init didn't complete successfully");

   }

  }

    public void update(Graphics g) {

      paint(g);

    }

    public void paint(Graphics g) {

      if (isWhite) {

       g.drawImage(offscreenBlack,0,0,this);

      } else {

       g.drawImage(offscreenWhite,0,0,this);

      }

      isWhite  =  !isWhite;

    }

   }

Silverlight animations were defined declaratively with XAML (eXtensible Application Markup Language). This content-oriented XML dialect allows defining declarative animations without specifying any details about their low-level implementation in Microsoft.Net. The XAML file included an animated Rectangle object loaded with the ColorAnimationUsingKeyFrames instruction with a duration of 1 sec (Duration = ”0∶0∶1.000”), repeating permanently (RepeatBehavior = ”Forever”) a white keyframe with a duration of 500 ms (KeyTime = ”0∶0∶0.500”). All tests are available for download at https://osf.io/6j3iz/.

### Results and discussion

**Figure 2:**
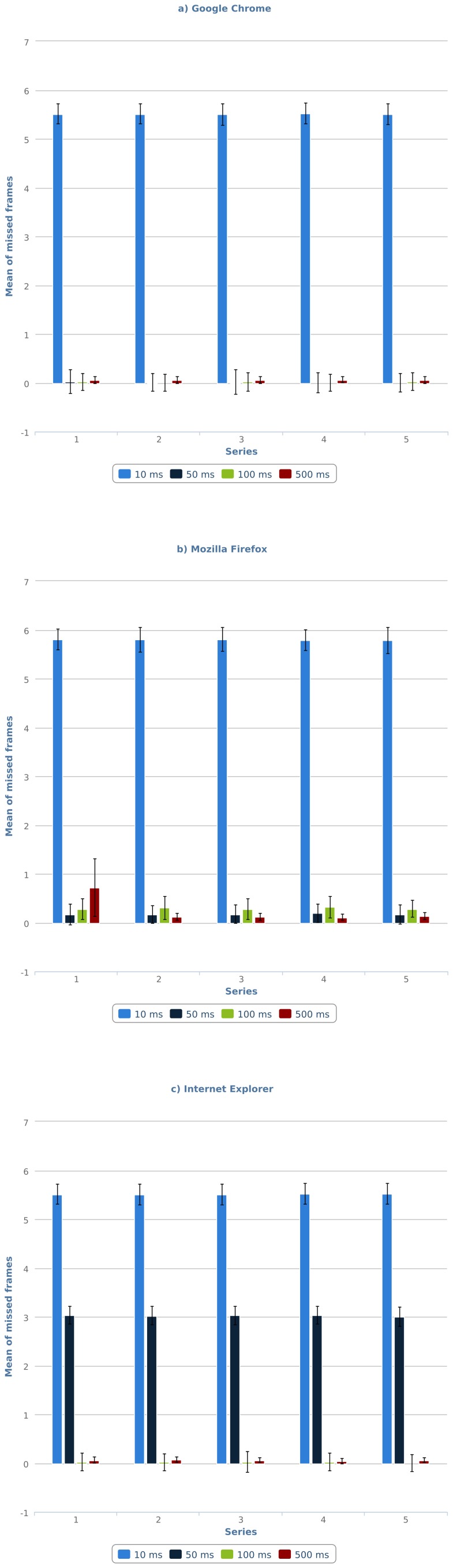
Number of missed frames per series for GIF89a animation on: (a) Google Chrome 17, (b) Mozilla Firefox 10, (c) Internet Explorer 9.

The number of missed frames in the GIF89a tests is shown in Table 1. As expected, this technology does not produce substantial delays when the animation interval is larger than 100 ms, regardless of the user-agent (Google Chrome 17, Mozilla Firefox 10, or Internet Explorer 9). Below that threshold, the resulting performance falls considerably, although unevenly, in all user-agents. In the case of Internet Explorer 9, any interval below 100 ms is reinterpreted as a 100-ms interval. Consequently, tests with 50 ms intervals show a 50 ms delay, whereas tests with 16.667 ms intervals show delays around 85 ms. In the case of Mozilla Firefox 10, GIF89a animations were executed correctly, except for the 16.667 ms interval, which yielded delays similar to those of Internet Explorer (around 85 ms). Finally, in the case of Google Chrome 17, the results of the tests with intervals below 100 ms are very similar to those observed in Mozilla Firefox 10, although the mean number of missed frames is smaller in all tests. Another interesting finding is that this technology is stable across time. As can be seen in Figure 2, the number of missed frames varies very little within the series of 5 consecutive tests for each case.

**Table 1:** Descriptive statistics of the number of missed frames for GIF89a animations.

	*500*		*100*		*50*		*10*	
**Google Chrome**								
Mean (SD)	0.06	(0.342)	0.01	(0.891)	0.01	(1.065)	5.51	(1.024)
Range	−2	+2	−1	+2	−2	+1	+4	+8
**Mozilla Firefox**								
Mean (SD)	0.24	(1.379)	0.29	(1.055)	0.17	(0.956)	5.79	(1.176)
Range	−1	+18	−1	+2	−2	+1	+4	+8
**Internet Explorer**								
Mean (SD)	0.06	(0.266)	0.02	(0.922)	3.02	(0.923)	5.51	(1.072)
Range	−1	+1	−3	+2	+1	+5	+3	+8

Regarding Adobe Flash, we evaluated the different setup and programming options offered by Adobe Flash to identify the best combination (see Study 1). After discarding the configurations that yielded the worst results, the performance of Adobe Flash was good, with few missed frames in each test (see Table 2).

**Table 2:** Descriptive statistics of the number of missed frames for Adobe Flash animations.

	*500*		*100*		*50*		*16.667*	
**Google Chrome**								
Mean (SD)	0.06	(0.234)	0.01	(0.109)	0.01	(0.425)	0.04	(0.215)
Range	0	+1	0	+1	−1	+2	0	+2
**Mozilla Firefox**								
Mean (SD)	0.06	(0.246)	0.01	(0.134)	0	(0.35)	0.05	(0.24)
Range	−1	+1	−1	+1	−1	+1	0	+2
**Internet Explorer**								
Mean (SD)	0.06	(0.238)	0.01	(0.134)	0.01	(0.816)	0.51	(0.653)
Range	0	+1	−1	+1	−1	+1	0	+2

The results of the tests conducted on Java are shown in Table 3. They were slightly worse than those obtained with Adobe Flash, especially regarding Google Chrome 17 and Internet Explorer 9 (the mean number of missed frames is larger than 0.8 in all setups).

**Table 3:** Descriptive statistics of the number of missed frames for Java animations.

	*500*		*100*		*50*		*16.667*	
**Google Chrome**								
Mean (SD)	0.94	(1.735)	0.85	(1.426)	1.71	(2.823)	1	(0.774)
Range	−6	+8	0	+8	0	+16	0	+5
**Mozilla Firefox**								
Mean (SD)	0.12	(0.325)	0.36	(1.845)	0.09	(0.514)	0.04	(0.249)
Range	0	+1	0	+13	0	+6	0	+2
**Internet Explorer**								
Mean (SD)	0.95	(2.057)	0.96	(1.695)	2.18	(3.182)	0.9	(0.443)
Range	−6	+8	0	+14	0	+16	0	+5

Table 4 shows the results of the tests conducted on Microsoft Silverlight. This technology behaved similarly to Adobe Flash, except for the 16.667 ms interval, which showed a worse performance in all user-agents (SDs above 1 in all cases).

**Table 4:** Descriptive statistics of the number of missed frames for Microsoft Silverlight animations.

	*500*		*100*		*50*		*16.667*	
**Google Chrome**								
Mean (SD)	0.06	(0.94)	0.01	(0.713)	0.02	(0.806)	0.26	(1.154)
Range	−3	+2	−2	+2	−2	+2	0	+13
**Mozilla Firefox**								
Mean (SD)	0.06	(0.893)	0.01	(0.741)	0.01	(0.797)	0.33	(1.533)
Range	−2	+2	−2	+2	−2	+2	0	+14
**Internet Explorer**								
Mean (SD)	0.06	(0.935)	0.02	(0.758)	0.01	(0.792)	0.28	(1.027)
Range	−2	+2	−2	+2	−2	+2	0	+11

To fully comprehend the pattern of results and to identify the most important factors of the number of missed frames, we conducted a 4 (Technology: GIF89a, Flash, Java, Silverlight) x 3 (User-Agent: Google Chrome 17, Mozilla Firefox, 10, Internet Explorer 9) x 4 (Interval: 500, 100, 50, 16.667) x 5 (Series: 1–5) x 2 (Colour: White vs. Black) ANOVA on the number of missed frames. As expected, the main effect of Series was not significant, indicating that performance does not change across the series of measurements (F(4, 23999) = 1.658, p = .157). As in our previous analyses, several main effects and interactions yielded significant results due to the relatively large number of data points that we recorded, even though their effect size was small. Among all the main effects analysed, the main effect of Interval is the most relevant in terms of effect size, F(4, 23999) = 6434.541, p<.001, η_p_^2^ = 0.523. Regarding the interactions between factors, Technology x User-Agent is the one with the largest effect size, F(16, 23999) = 82.384, p<0.001, η_p_^2^ = 0.053. Note, however, the relatively small effect size of this interaction. Therefore, the performance of these technologies might vary depending on the time interval and the specific combination of technology and user-agent. All other main effects and interactions had very small effect sizes (all η_p_^2^s<0.05).

Overall, the accuracy and precision in the presentation of visual content in classic web technologies was acceptable, with a mean number of missed frames below 1 in most tests where intervals lasted above 100 ms, and standard deviations ranging from 0.109 (100-ms Flash animations on Google Chrome) to 2.057 missed frames (500-ms Java animations on Internet Explorer).

However, some results suggest that in certain conditions, these technologies should be used with caution. In the case of GIF89a, the results are good for intervals equal or larger than 50 ms in Google Chrome 17 and Mozilla Firefox 10, and also for intervals equal or larger than 100 ms in Internet Explorer. But these animations cannot control or synchronize their status once they have been started. Consequently, this technology is not adequate for many web applications that require high accuracy and precision in the presentation of multimedia content.

Flash, possibly the most popular current option for the development of interactive web applications, yielded very good results. In all cases, except in Internet Explorer 9 with 16.667 ms, it produced a mean number of missed frames below 0.1 and a standard deviation below 1. These values were obtained using the loop timing mechanism, since the mechanisms implemented in ActionScript (setInterval, polling, and timer) yielded worse results in our previous tests.

Regarding Java, the poor performance observed in Google Chrome 17 and Internet Explorer 9 (around 1 missed frame, on average, in all conditions) contrasts the good results obtained in Mozilla Firefox 10 (on average, below 0.4 missed frames in all conditions). This result is surprising, since Java is a technology based on a virtual machine with reduced interaction with the HTML document where it is embedded.

Finally, despite its reduced popularity among users and the low number of web applications designed for Silverlight, its performance is very similar to that of Flash. The exception is noticeably worse accuracy and precision in tests with the shortest interval (16.667 ms), which is reasonable for a client-based technology that was developed as an analogue of Adobe Flash.

## Study 3: HTML5 web technologies

The aim of Study 3 is to conduct a comprehensive analysis of the precision and accuracy of the animations created using combinations of modern (HTML5-related) web technologies. First, we analysed web technologies to create declarative animations: CSS Animations and SVG with SMIL. Next, we analysed web technologies to create procedural animations: SVG with JavaScript, Canvas and WebGL. In all procedural animations tests, the API for the Temporal Control of Procedural Animations (i.e., requestAnimationFrame) was used in order to avoid the problems related to the use of standard JavaScript timers (setTimeout and setInterval).

### Methodology and apparatus

In this study we used the same hardware as in previous studies. We installed three different operating systems in the computer described above: 1) Microsoft Windows 7 Professional 32-bit edition with Service Pack 1; 2) Ubuntu Linux 10.04 LTS “Lucid Linx” 32-bit edition; and 3) Apple Mac OS X 10.7.3 “Lion”. We chose these because they were the most stable versions of each operating system at the time the tests were conducted. We used two user-agents compatible with the three operating systems: Google Chrome and Mozilla Firefox. In each case, we used the latest version that was available when the study was conducted (Google Chrome 17 and Mozilla Firefox 10).

### Procedure

We again defined non-gradual black-to-white keyframe transitions, varying the duration of each keyframe with values 500, 100, 50, and 16.667 ms (i.e., 30, 6, 3, and 1 tick at 60 Hz, respectively). All the tests consisted of a 200×200 pixel animation placed at the centre of the screen. In each case, we recorded five independent 60-second series, but analysed only the first 100 samples of each series. Therefore, 500 samples were recorded for each combination of interval (500, 100, 50, and 16.667 ms), web technology (CSS Animations, SVG with SMIL, SVG with JavaScript, Canvas, and WebGL), user-agent (Google Chrome, and Mozilla Firefox) and operating system (Microsoft Windows, MacOS X, GNU/Linux).

We tested declarative animations in HTML5 using CSS Animations and SVG with SMIL, and prepared procedural animations were prepared with SVG, Canvas, and WebGL with JavaScript. In all cases, we used the timer provided by the API for the Temporal Control of Procedural Animations (i.e., requestAnimationFrame). These technologies were tested in Google Chrome 17 and Mozilla Firefox 10 running under Microsoft Windows 7 SP 1, Ubuntu Linux 10.04 LTS, and Apple Mac OS X 10.7. We selected these user-agents because both of them were available for the three operating systems and because they were compatible with all the technologies mentioned so far.

CSS animations were defined using two keyframes. The first (run at keyframe 0%, which indicates the first moment of the animation sequence) sets the background colour of the div element to white and the second (run at keyframe 50%), sets it back to black. The total duration of the animation is twice the value of the tested interval. Thus, for a 100 ms test, a 200 ms duration is defined, so that 50% of the total duration of the animation corresponds to the desired 100 ms keyframe duration. Given that a non-gradual transition from black to white was required, we used the step function for transitions. In the case of SVG with SMIL, it was necessary to prepare two different versions for each user-agent, because Google Chrome 17 does not support cross-references in the definition of an animation's onset. Because of this, we unrolled the animation loops for SVG with SMIL tests in this user-agent using shell scripts. These SVG files do not therefore loop the animation endlessly, but perform a fixed number of changes (e.g., 120 changes for a 1-minute animation with an interval rate of 500 ms). Procedural animations all use a similar structure based on requestAnimationFrame, that updates the background colour of the object with the required methods in each case. Therefore, our tests of the accuracy and precision of the Canvas 2D API to present visual content rely on the fillStyle property and the fillRect method to update the background colour of the animation. In the case of WebGL, the background colour is updated with clearcolor and clear methods of context 3D. This was done using the setAttributeNS method in SVG procedural animations. All tests are available for download at https://osf.io/6j3iz/.

### Results and discussion

Table 5 shows the results of the tests conducted using CSS. There is a marked decline in performance in tests with intervals below 50 ms, with particularly poor results for Mozilla Firefox 10 on Windows 7 (M: 4.89, SD: 5.041). Above that interval, performance is comparable with the results of classic online technologies.

**Table 5:** Descriptive statistics of the number of missed frames for CSS animations.

		*500*		*100*		*50*		*16.667*	
**Windows7**									
	**Google Chrome**								
	Mean (SD)	0.06	(0.649)	0.01	(0.522)	0	(0.606)	2.62	(0.79)
	Range	−2	+2	−2	+1	−2	+2	+2	+7
	**Mozilla Firefox**								
	Mean (SD)	0.06	(0.603)	0.01	(1.043)	0.01	(1.107)	4.89	(5.041)
	Range	−2	+2	−3	+2	−2	+3	0	+22
**GNU/Linux**									
	**Google Chrome**								
	Mean (SD)	0	(0.961)	0	(0.537)	0	(0.459)	2.17	(0.588)
	Range	−2	+2	−2	+2	−2	+2	0	+4
	**Mozilla Firefox**								
	Mean (SD)	0	(0.708)	0	(0.655)	0	(0.789)	0.32	(0.691)
	Range	−2	+2	−2	+2	−2	+2	0	+3
**Mac OS X**									
	**Google Chrome**								
	Mean (SD)	−0.06	(0.85)	0	(0.667)	0	(0.461)	2.42	(0.71)
	Range	−1	+1	−2	+1	−2	+1	+2	+4
	**Mozilla Firefox**								
	Mean (SD)	−0.06	(0.707)	−0.01	(0.668)	0	(0.537)	0.14	(0.362)
	Range	−1	+1	−1	+1	−1	+1	0	+3

**Figure 3:**
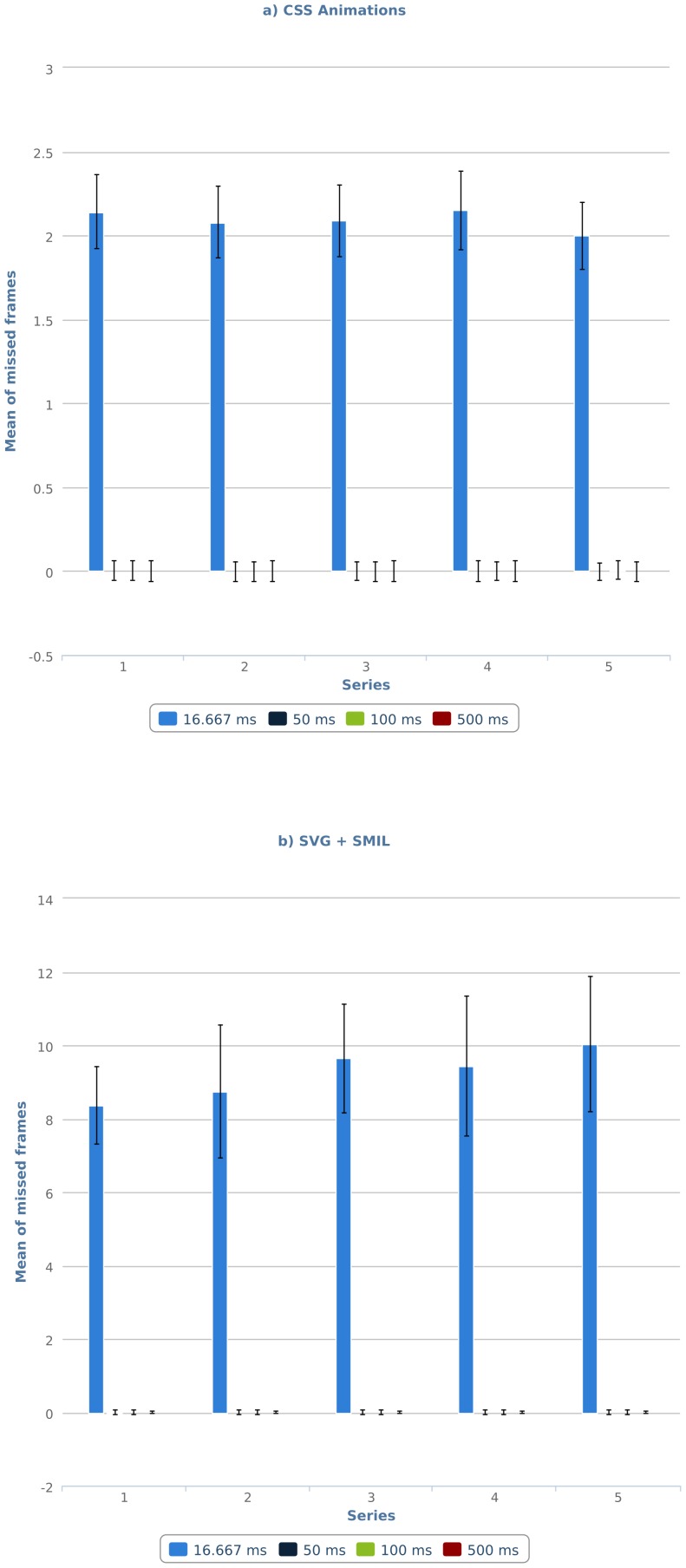
Number of missed frames per series for: (a) CSS animations, (b) SVG+ SMIL animations.

The tests of SVG and SMIL yielded a pattern of results very similar to that observed in tests of CSS Animations (see Table 6). However, the decline in performance in tests with intervals below 50 ms is more pronounced, producing extremely poor results in Google Chrome 17 under Windows 7 (M: 24.69, SD: 44.167). Overall, the performance of both technologies for declarative animations is equal or better than the one observed in the equivalent tests of classic web technologies, except in the case of 16.667 ms, where the performance falls to unacceptable levels. As can be seen in Figure 3, these values remain stable, except for condition 16.667 in SVG and SMIL, which suffers a noticeable increase in the number of missed frames.

**Table 6:** Descriptive statistics of the number of missed frames for SVG+SMIL animations.

		*500*		*100*		*50*		*16.667*	
**Windows7**									
	**Google Chrome**								
	Mean (SD)	0.06	(0.281)	0.01	(0.893)	0.01	(1.041)	24.69	(44.167)
	Range	−2	+2	−2	+2	−2	+1	0	498
	**Mozilla Firefox**								
	Mean (SD)	0.07	(0.575)	0.01	(1.092)	0	(1.082)	4.73	(4.055)
	Range	−1	+2	−3	+2	−2	+3	0	+16
**GNU/Linux**									
	**Google Chrome**								
	Mean (SD)	0	(0.11)	0	(0.564)	0	(0.753)	11.11	(3.983)
	Range	−1	+1	−1	+2	−2	+1	0	+39
	**Mozilla Firefox**								
	Mean (SD)	0	(0.729)	0	(0.65)	0	(0.806)	0.34	(0.719)
	Range	−3	+4	−2	+2	−2	+2	0	+3
**Mac OS X**									
	**Google Chrome**								
	Mean (SD)	−0.06	(0.254)	−0.01	(0.257)	0	(0.393)	14.47	(4.937)
	Range	−1	+1	−1	+1	−1	+1	+1	+40
	**Mozilla Firefox**								
	Mean (SD)	−0.06	(0.705)	−0.01	(0.679)	0	(0.554)	0.13	(0.369)
	Range	−1	+1	−1	+1	−1	+1	0	+2

The tests of procedural animations with the Canvas 2D API yielded similar results in all conditions, with means ranging from 0.11 to 0.94 missed frames, and standard deviations from 0.265 to 1.055 (see Table 7). A similar pattern is observed in SVG and JavaScript animations, with means ranging from 0.07 to 1.50 and standard deviations from 0.252 to 0.890 (see Table 8), and in WebGL animations, with means between 0.09 and 0.98 and standard deviations between 0.262 and 1.028 (see Table 9). In all cases, some specific combinations of user-agent and operating systems yielded particularly good results (e.g., Mozilla Firefox 10 under Mac Os X is the most successful combination in most tests). The factors responsible for the worst performance are more difficult to identify, given that the worst results are scattered unevenly over different user-agents and explorers in each test.

**Table 7:** Descriptive statistics of the number of missed frames for Canvas 2D animations.

		*500*		*100*		*50*		*16.667*	
**Windows7**									
	**Google Chrome**								
	Mean (SD)	0.8	(0.399)	0.85	(0.547)	0.85	(0.557)	0.93	(0.504)
	Range	0	+1	0	+2	0	+2	0	+3
	**Mozilla Firefox**								
	Mean (SD)	0.66	(0.7)	0.69	(1.055)	0.85	(0.579)	0.94	(0.468)
	Range	0	+3	−1	+2	0	+3	0	+3
**GNU/Linux**									
	**Google Chrome**								
	Mean (SD)	0.11	(0.708)	0.8	(0.467)	0.88	(0.442)	0.82	(0.726)
	Range	−2	+1	−1	+3	−1	+2	0	+3
	**Mozilla Firefox**								
	Mean (SD)	0.76	(0.556)	0.18	(0.446)	0.09	(0.589)	0.34	(0.685)
	Range	−1	+3	−2	+2	−2	+2	0	+4
**Mac OS X**									
	**Google Chrome**								
	Mean (SD)	0.54	(0.507)	0.48	(0.516)	0.35	(0.493)	0.56	(0.497)
	Range	−1	+1	−1	+1	−1	+1	0	+1
	**Mozilla Firefox**								
	Mean (SD)	0.45	(0.498)	0.47	(0.499)	0.22	(0.412)	0.06	(0.265)
	Range	0	+1	0	+1	0	+1	0	+3

**Table 8:** Descriptive statistics of the number of missed frames for SVG+JavaScript animations.

		*500*		*100*		*50*		*16.667*	
**Windows7**									
	**Google Chrome**								
	Mean (SD)	0.8	(0.397)	0.85	(0.548)	0.85	(0.567)	0.93	(0.485)
	Range	0	+1	0	+3	−1	+2	0	+3
	**Mozilla Firefox**								
	Mean (SD)	1.3	(0.89)	1.5	(0.5)	0.76	(0.466)	0.88	(0.33)
	Range	0	+4	+1	+2	0	+3	0	+1
**GNU/Linux**									
	**Google Chrome**								
	Mean (SD)	0.11	(0.677)	0.8	(0.508)	0.88	(0.401)	0.81	(0.738)
	Range	−2	+2	−1	+3	−1	+3	0	+3
	**Mozilla Firefox**								
	Mean (SD)	0.77	(0.647)	0.18	(0.446)	0.09	(0.564)	0.35	(0.753)
	Range	−1	+3	−1	+2	−2	+2	0	+4
**Mac OS X**									
	**Google Chrome**								
	Mean (SD)	0.56	(0.508)	0.5	(0.512)	0.4	(0.498)	0.53	(0.503)
	Range	−1	+1	−1	+1	−1	+1	0	+2
	**Mozilla Firefox**								
	Mean (SD)	0.54	(0.499)	0.52	(0.5)	0.26	(0.437)	0.07	(0.252)
	Range	0	+1	0	+1	0	+1	0	+1

**Table 9:** Descriptive statistics of the number of missed frames for WebGL animations.

		*500*		*100*		*50*		*16.667*	
**Windows7**									
	**Google Chrome**								
	Mean (SD)	0.78	(0.413)	0.73	(0.443)	0.85	(0.359)	0.93	(0.262)
	Range	0	+1	0	+1	0	+1	0	+1
	**Mozilla Firefox**								
	Mean (SD)	0.98	(0.942)	0.69	(1.028)	0.91	(0.659)	0.95	(0.514)
	Range	0	+3	−1	+2	0	+3	0	+3
**GNU/Linux**									
	**Google Chrome**								
	Mean (SD)	0.3	(0.462)	0.84	(0.369)	0.9	(0.295)	0.81	(0.396)
	Range	−1	+1	0	+1	0	+1	0	+1
	**Mozilla Firefox**								
	Mean (SD)	0.73	(0.536)	0.17	(0.486)	0.09	(0.353)	0.4	(0.725)
	Range	−1	+2	−1	+2	−2	+2	0	+3
**Mac OS X**									
	**Google Chrome**								
	Mean (SD)	0.58	(0.507)	0.47	(0.523)	0.44	(0.497)	0.55	(0.498)
	Range	−1	+1	−1	+2	0	+1	0	+1
	**Mozilla Firefox**								
	Mean (SD)	0.46	(0.608)	0.3	(0.471)	0.12	(0.34)	0.09	(0.32)
	Range	−5	+6	−1	+1	−1	+2	0	+3

To get a clearer view of the factors responsible for performance in these tests, we conducted a 5 (Technology: CSS, SMIL, SVG, Canvas, WebGL) x 2 (User-Agent: Google Chrome 17, Mozilla Firefox 10) x 3 (Operating system: Windows 7, GNU/Linux, Mac OS X) x 4 (Interval: 500, 100, 50, 16.667) x 5 (Series: 1-5) x 2 (Colour: White vs. Black) ANOVA on the number of missed frames. The results of this analysis are very similar to the previous one: 1) as a result of the large number of observations, many effects reach significance levels; 2) once more, the main effect of Series is not significant, showing that these technologies do not suffer from temporal degradation, F<1; 3) among all the main effects analysed, the main effect of Interval is the most relevant in terms of effect size, F(3, 59999) = 1127.896, p<0.001, η_p_^2^ = 0.054. The most relevant interaction is Technology x Interval, F(24, 59999) = 695.966, p<0.001, η_p_^2^ = 0.124, probably due to the poor results drawn by CSS and SMIL in 16.667 ms animations. These results are consistent with the previous interpretation of the differences found across intervals, user-agents, and operating systems. However, even these two effects have a very small size. All the datasets regarding these results are available to download at https://osf.io/6j3iz/.

We conclude that, among all the tested technologies, declarative animations based on CSS are the most effective alternative when animation intervals are above 50 ms, given that they yielded a low number of missed frames (means from 0.00 to −0.06, standard deviations from 0.459 to 1.107). They are also temporally stable (without a main effect of Series or important interactions involving this factor) and independent of the performance of JavaScript (it does not overload its event queue and it is not affected by it, either). The same conclusion could be extended to the combination of SVG and SMIL, except for their inefficient implementation in Google Chrome, which precludes a good performance and does not allow cross-referenced temporal loops.

The performance of procedural web technologies around the HTML5 standard (Canvas, SVG, an WebGL with requestAnimationFrame) is very similar to that of previously analysed web technologies (means of missed frames between 0.06 and 1.50 with standard deviations between 0.252 and 1.055, compared to means between 0.00 and 2.18 and standard deviations between 0.109 and 3.182). Furthermore, these technologies are becoming standard and have a promising future ahead, which makes their use clearly more advisable overother technologies whose use is in clear decline (Java, Flash, Silverlight) [9].

## Conclusions and Outlook

Even though the accuracy and precision of HTML5 technologies still has room for improvement, we can conclude that the continuous development in the performance of the new web standards related to HTML5 indicates a promising future for web applications that require accurate and precise presentation of visual content.

### Implications for Web-based research

As mentioned in the Introduction, the results of this study have implications for current attempts to implement behavioural and social studies in online technologies. Some popular experimental paradigms require the accurate and precise presentation of very brief visual stimuli. For instance, experiments on subliminal priming usually require presenting stimuli with durations between 16 and 100 ms [4], [5]. Some of these effects have been replicated using Web technologies [27]. However, our results show that not all web technologies are equally valid to develop applications with these strict time constraints. Given the variable performance of user-agents, timers, and technologies, researchers should carefully decide which technologies to use, depending on the requirements of their experiments. They should also report the specific details of the timing mechanisms in the Methods section of their paper. Without this information, their findings might not be replicable by other researchers relying on similar, but not identical, implementations.

Although the present study focuses on the accuracy and precision of stimuli durations, our results are also relevant for the measurement of reaction times in Web-delivered experiments. Reaction times are quickly becoming a common dependent variable in psychological experiments conducted over the Internet [6], [29]. However, the accuracy in the timing of stimuli imposes a limit in the accuracy in the registration of reaction times: if a stimulus has not been presented at the exact time or with the exact duration, the actual reaction time of participants might not be correctly measured. Therefore, researchers interested in collecting reaction times in Web-delivered experiments should make sure that they are using the most accurate technologies for the timing of stimuli.

### Limitations of present research

The current study has been an attempt to analyse all the possibilities offered by new web technologies to create precise and accurate animations. However, the huge amount of tests needed to carry out this objective, together with the rapid development cycles of the technologies used, make difficult, if not impossible, to offer results and conclusions about the latest versions of those technologies. The competition among the main developers of user-agents (Google, Microsoft, Mozilla, Apple, Opera) has resulted in a plethora of updates, which are hard to keep current (e.g., Mozilla Firefox took longer than seven years to pass from version 1.0 to 5.0, but in the last three years has published over twenty-five new versions).

Something similar is happening at a slower pace with operating systems which have short release publication periods, e.g. Ubuntu Linux. Given the new release pace of browsers and operating systems, researchers will inevitably lag behind and publish results and conclusions derived from out-of-date software. Nevertheless, research performed over the Internet delegates the software update responsibility to the participant in the experiment. For this reason, the results presented in this study are valuable and useful not only for those researchers who use these technologies only in their labs, but also for researchers who conduct experiments on the Internet.

Ideally, someone would publish an open access database constantly updated with evidences about the precision and accuracy of web technologies used for online research. Our contribution to that envisaged database is already published in the Open Science Framework (OSF). Both the tests and their results can be downloaded from our OSF repository.

A second issue has to do with the hardware in which the tests have taken place. In previous studies (see [18]; but particularly [19]), the software analysed was tested over different hardware systems to assess the variability of results between-systems. Taking into account the little control that researchers carrying out Internet research have on the hardware used by the participants in their experiments, it is valuable to have data about the variability among hardware systems. Our study focused on software, undertaking an ample comparison of a wide range of technologies among them, over an ample number of combinations of user-agents and operating systems. Studies such as the one from Reimers and Stewart [20] complement this one to offer Internet researchers an insight into which technologies are most suitable for each type of experiment. Hopefully, other researchers will continue our work and analyse these software technologies on different hardware equipment in order to collaboratively contribute to the public, open access database mentioned in the previous paragraph.

Even having such open continuously updated database including information about the precision and accuracy of the web technologies, it is important to recall that following the recommendations of this study does not prevent researchers from conducting their own tests in their experimental setups [30]. The purpose of the analysis presented here is to guide researchers towards which web technologies are appropriate for a given study. After selecting one of these technologies, it is the responsibility of researchers to verify the accuracy and precision of their tools in a variety of scenarios that represent plausible variations of hardware, software and network connectivity that may occur among their participants.

## References

[1] Birnbaum MH (2004) Human research and data collection via the Internet. Annu Rev Psychol, 55, 803–832. 14744235 10.1146/annurev.psych.55.090902.141601

[2] Schmidt W (2001) Presentation accuracy of Web animation methods. Beh Res Methods, 33(2): 187–200. 10.3758/bf03195365 11447672

[3] Eichstaedt J (2001) An inaccurate-timing filter for reaction time measurement by Java applets implementing Internet-based experiments. Beh Res Methods, 33(2): 179–186. 10.3758/bf03195364 11447671

[4] Dehaene S, Naccache L, Le Clec'H G, Koechlin E, Mueller M, et al (1998) Imaging unconscious semantic priming. Nature, 395: 597–600. 9783584 10.1038/26967

[5] Hassin RR, Ferguson MJ, Shidlovski D, Gross T (2007) Subliminal exposure to national flags affects political thought and behavior. Proc Natl Acad Sci U S A, 104: 19757–19761. 18056813 10.1073/pnas.0704679104 PMC2148371

[6] Nosek BA (2005) Moderators of the relationship between implicit and explicit evaluation. J Exp Psychol: General, 134, 565–584. 10.1037/0096-3445.134.4.565 PMC1440676 16316292

[7] Voss A, Rothermund K, Gast A, Wentura D (2013) Cognitive processes in associative and categorical priming: A diffusion model analysis. J Exp Psychol: General, 142: 536–559. 10.1037/a0029459 22866687

[8] CompuServe (1990) Graphics Interchange Format programming reference. Available: http://www.w3.org/Graphics/GIF/spec-gif89a.txt

[9] W3Techs (2014) Usage of Java for websites. Available: http://w3techs.com/technologies/details/cp-javaruntime/all/all

[10] Goodin D (2013) Critical Java zero-day bug is being “massively exploited in the wild”. Ars Technica. Available: http://arstechnica.com/security/2013/01/critical-java-zero-day-bug-is-being-massively-exploited-in-the-wild/

[11] Jobs S (2010) Thoughts on Flash. Available: https://www.apple.com/hotnews/thoughts-on-flash/

[12] Winokur D (2011) Flash to focus on PC browsing and mobile apps; Adobe to more aggressively contribute to HTML5. Available: http://blogs.adobe.com/conversations/2011/11/flash-focus.html

[13] Chappell D (1996) Understanding ActiveX and OLE: a guide for developers and managers. Microsoft Press.

[14] Oeschger I (2002) API reference: Netscape Gecko plugins. Netscape Communications.

[15] Yee B, Sehr D, Dardyk G, Chen J, Muth R, et al.. (2009) Native client: A sandbox for portable, untrusted x86 native code. In 30th IEEE Symposium on Security and Privacy (pp. 79–93).

[16] Resig J (2008) Accuracy of JavaScript Time. Available: http://ejohn.org/blog/accuracy-of-javascript-time/

[17] Jackson D, Hyatt D, Marrin C, Galineau S, Baron D (2013) CSS animations. W3C Working Draft, 19 February 2013. Available: http://www.w3.org/TR/css3-animations/

[18] Adams C (2010) HTML5 versus Flash: Animation benchmarking. Available: http://www.themaninblue.com/writing/perspective/2010/03/22/

[19] Neath I, Earle A, Hallett D, Surprenant AM (2011) Response time accuracy in Apple Macintosh computers. Beh Res Methods, 43: , 353–362. 10.3758/s13428-011-0069-9 21416303

[20] Reimers S, Stewart N (2014) Presentation and response timing accuracy in Adobe Flash and HTML5/JavaScript Web experiments. Beh Res Methods, doi:10.3758/s13428-014-0471-1 10.3758/s13428-014-0471-1 PMC4427652 24903687

[21] Keller F, Gunasekharan S, Mayo N, Corley M (2009) Timing accuracy of web experiments: A case study using the Webexp software package. Beh Res Methods, 41(1): , 1–12. 10.3758/BRM.41.1.12 19182118

[22] McGraw K, Tew M, Williams J (2000) The integrity of web-delivered experiments: Can you trust the data? Psychol Sci, 11(6): , 502–506. 10.1111/1467-9280.00296 11202497

[23] Reimers S, Stewart N (2007) Adobe flash as a medium for online experimentation: A test of reaction time measurement capabilities. Beh Res Methods, 39(3): , 365–370. 10.3758/bf03193004 17958146

[24] Stewart N (2006) Millisecond accuracy video display using OpenGL under Linux. Beh Res Methods, 38(1): , 142–145. 10.3758/bf03192759 16817523

[25] Reimers S, Stewart N (2008) Using Adobe Flash Lite on mobile phones for psychological research: Reaction time measurement reliability and interdevice variability. Beh Res Methods, 40(4): , 1170–1176. 10.3758/BRM.40.4.1170 19001409

[26] Schubert TW, Murteira C, Collins EC, Lopes D (2013) ScriptingRT: A software library for collecting response latencies in online studies of cognition. PLoS ONE, 8: , e67769. doi:10.1371/journal.pone.0067769 10.1371/journal.pone.0067769 PMC3689727 23805326

[27] Crump MJ, McDonnell JV, Gureckis TM (2013) Evaluating Amazon's Mechanical Turk as a tool for experimental behavioral research. PLoS ONE, 8: , e57410 10.1371/journal.pone.0057410 PMC3596391 23516406

[28] Plant R, Hammond N, Turner G (2004) Self-validating presentation and response timing in cognitive paradigms: How and why? Beh Res Meth Instr C, 36(2): , 291–303. 10.3758/bf03195575 15354695

[29] Garaizar P, Vadillo MA, López-de-Ipiña D, Matute H (2014) Measuring software timing errors in the presentation of visual stimuli in cognitive neuroscience experiments. PLoS ONE 9(1): e85108 doi:10.1371/journal.pone.0085108 24409318 10.1371/journal.pone.0085108 PMC3883681

[30] Plant RR, Quinlan PT (2013) Could millisecond timing errors in commonly used equipment be a cause of replication failure in some neuroscience studies? Cogn Affect Behav Ne, 13: , 598–614. 10.3758/s13415-013-0166-6 23640111

